# Role of Licochalcone A in Potential Pharmacological Therapy: A Review

**DOI:** 10.3389/fphar.2022.878776

**Published:** 2022-05-23

**Authors:** Meng-Ting Li, Long Xie, Hai-Mei Jiang, Qun Huang, Rong-Sheng Tong, Xiang Li, Xin Xie, Hong-Mei Liu

**Affiliations:** ^1^ Department of Pharmacy, Personalized Drug Therapy Key Laboratory of Sichuan Province, Sichuan Provincial People’s Hospital, School of Medicine, University of Electronic Science and Technology of China, Chengdu, China; ^2^ State Key Laboratory of Southwestern Chinese Medicine Resources, Hospital of Chengdu University of Traditional Chinese Medicine, School of Pharmacy and College of Medical Technology, Chengdu University of Traditional Chinese Medicine, Chengdu, China

**Keywords:** flavonoid, licochalcone A, anticancer, pharmacological therapy, traditional Chinese medicine

## Abstract

Licochalcone A (LA), a useful and valuable flavonoid, is isolated from *Glycyrrhiza uralensis* Fisch. ex DC. and widely used clinically in traditional Chinese medicine. We systematically updated the latest information on the pharmacology of LA over the past decade from several authoritative internet databases, including Web of Science, Elsevier, Europe PMC, Wiley Online Library, and PubMed. A combination of keywords containing “Licochalcone A,” “Flavonoid,” and “Pharmacological Therapy” was used to help ensure a comprehensive review. Collected information demonstrates a wide range of pharmacological properties for LA, including anticancer, anti-inflammatory, antioxidant, antibacterial, anti-parasitic, bone protection, blood glucose and lipid regulation, neuroprotection, and skin protection. LA activity is mediated through several signaling pathways, such as PI3K/Akt/mTOR, P53, NF-κB, and P38. Caspase-3 apoptosis, MAPK inflammatory, and Nrf2 oxidative stress signaling pathways are also involved with multiple therapeutic targets, such as TNF-α, VEGF, Fas, FasL, PI3K, AKT, and caspases. Recent studies mainly focus on the anticancer properties of LA, which suggests that the pharmacology of other aspects of LA will need additional study. At the end of this review, current challenges and future research directions on LA are discussed. This review is divided into three parts based on the pharmacological effects of LA for the convenience of readers. We anticipate that this review will inspire further research.

## Introduction

**FIGURE 1:**
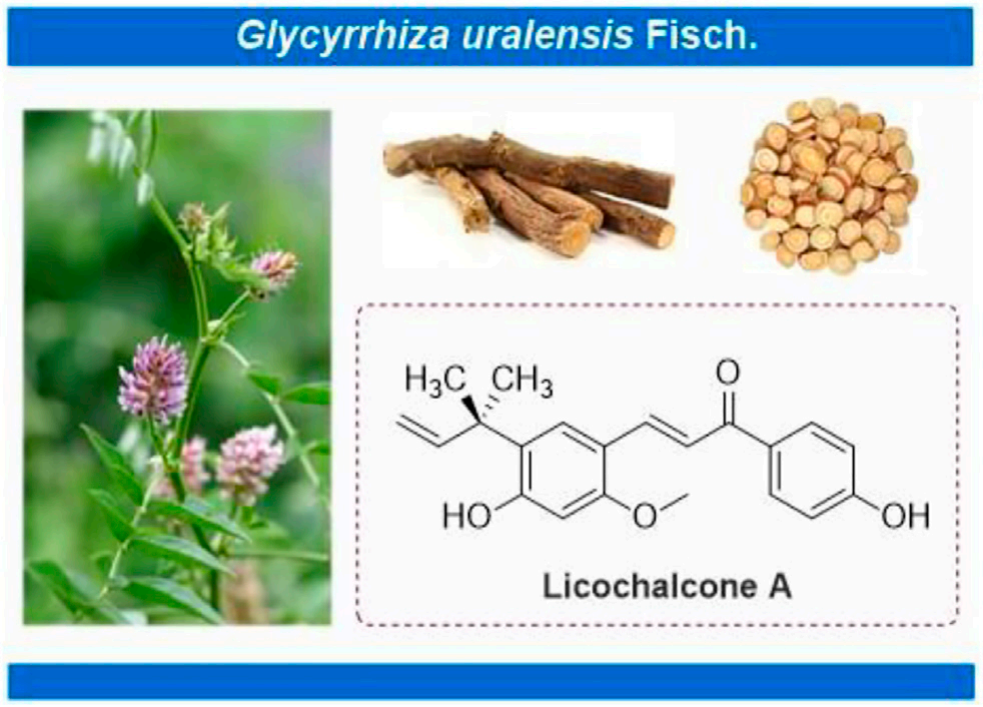
Licochalcone A isolated from *Glycyrrhiza uralensis* Fisch. ex DC.

**FIGURE 2:**
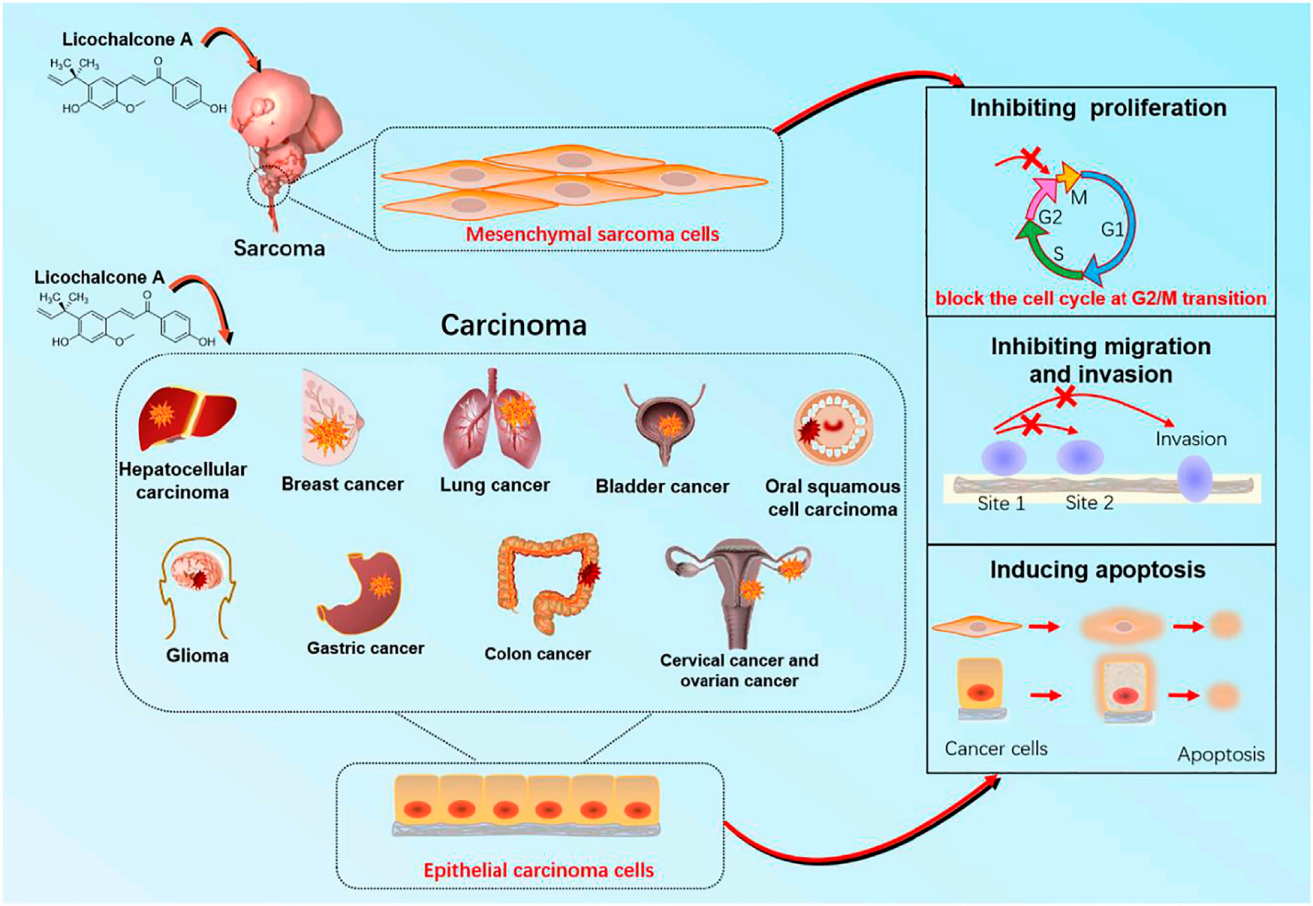
The target organ and cellular mechanisms for anticancer effect of licochalcone A with anticancer activity.

Licochalcone A (LA) (CAS No: 58749-22-7), with a molecular formula of C_21_H_22_O_4_, is a valuable flavonoid primarily extracted from roots of *Glycyrrhiza uralensis* Fisch. ex DC. (Figure 1) (Simmler et al., 2017; Wang et al., 2020). Licorice always functions as an adjuvant drug in traditional Chinese medicine to reduce the toxicity of other medicinal herbs or enhance their pharmacological effects. LA, thus, helps achieve the treatment or prevention of complex diseases (Jiang et al., 2020). Licorice demonstrates unique medicinal properties for clearing heat and toxins in clinical practice. LA has, thus, attracted the attention of pharmacologists worldwide (Jiang et al., 2020). Significant advancements have been witnessed in pharmacological research over the past decades. LA inhibits the proliferation of epithelial carcinoma and mesenchymal sarcoma cells. Examples include hepatocellular carcinoma, breast and colon cancer of epithelial origin, and melanoma of mesenchymal origin (Figure 2). Furthermore, LA demonstrates various pharmacological properties, including anti-inflammation, antibacterial, antioxidant, anti-parasitic, bone protection, neuroprotection, skin protection, and blood glucose and lipid regulation. The significant potential of LA for the treatment and prevention of clinical diseases indicates that an updated review regarding the therapeutic pharmacology of LA is timely. Therefore, we summarize the latest advancements for understanding the pharmacology of LA and explore the potential of this unique bioactive compound for use in the treatment of various diseases. We further provide useful and fundamental information for LA’s rational and secure application. This review is divided into three parts, 1) anticancer activities, 2) anti-inflammation activities, and 3) other pharmacological activities, for the convenience of readers.

## Anticancer Properties of LA

**FIGURE 3:**
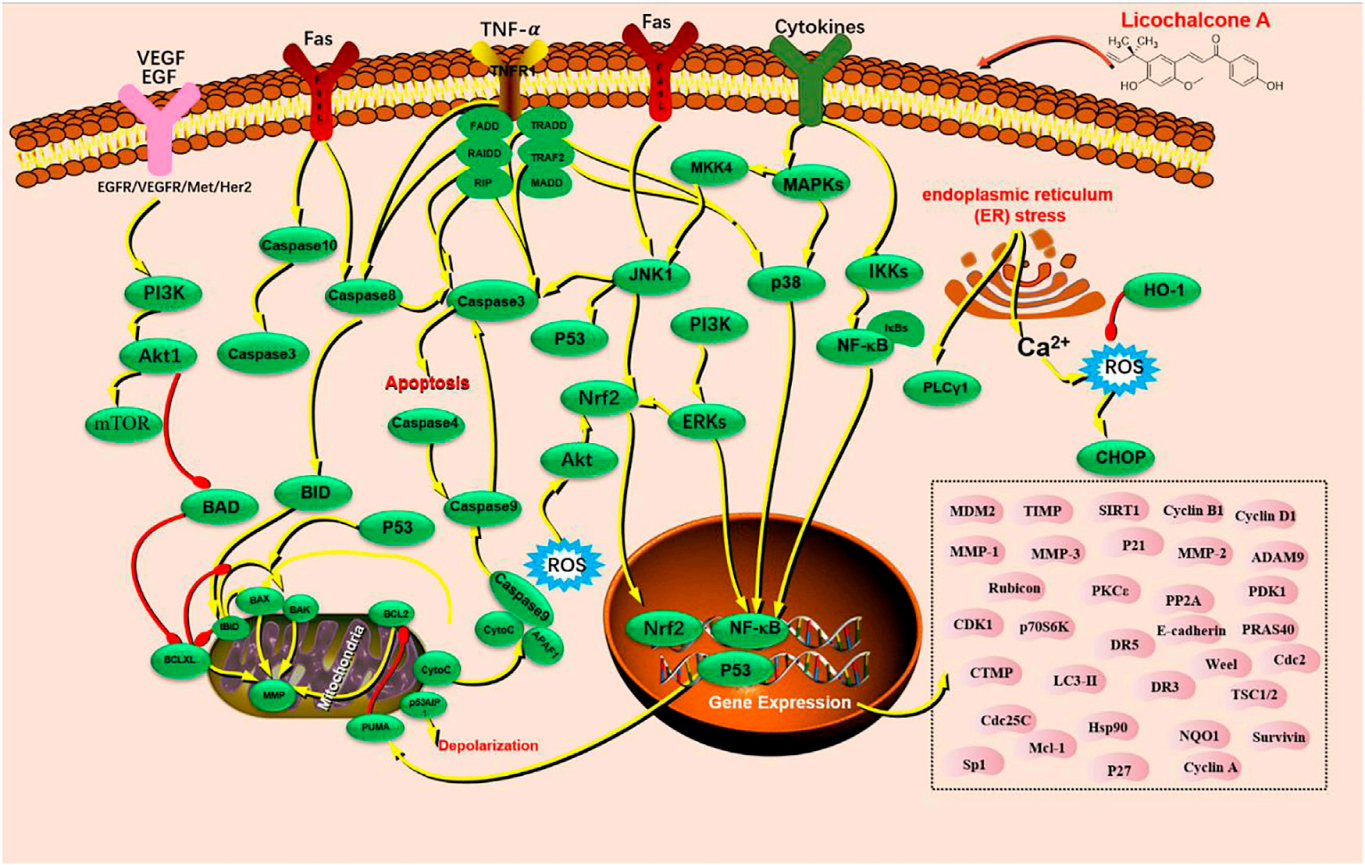
The signaling pathways of licochalcone A with anticancer activity.

Cancers are major malignant diseases worldwide that seriously threaten human health and life. The significant feature of cancer is the rapid growth of abnormal cells that extends beyond their usual boundaries, invade adjoining tissues, and spread to other organs. These processes are termed proliferation, invasion, and migration (Kennedy and Salama, 2020; Siegel et al., 2021). The anticancer activity of LA is mainly associated with epithelial carcinoma and mesenchymal sarcoma cells. Cancers mediated by epithelial carcinoma cells include hepatocellular, glioma, breast, gastric, colon, lung, cervical, ovarian, bladder, and oral squamous cell. Melanoma is primary cancer caused by mesenchymal sarcoma cells (Figure 2). LA is mainly involved in cellular processes such as apoptosis and endoplasmic reticulum (ER) stress, which underlie anticancer activity. LA activity is mediated *via* several signaling pathways, such as PI3K/Akt/mTOR, P53, NF-κB, and P38. Caspase-3 apoptosis, MAPK inflammatory, and Nrf2 oxidative stress signaling are also involved along with multiple therapeutic targets, including TNF-α, VEGF, Fas, FasL, PI3K, AKT, caspase-3, caspase-4, caspase-8, caspase-9, caspase-10, P38, P53, BAX, BAD, BID, JNK1, IL-1β, IL-8, ECM, SOD, IFN-γ, IKK, HO-1, MAPK, and ERK (Figure 3).

### Effects of LA on Inducing Apoptosis of Cancer Cells

As an effective pharmacological compound for cancer, LA induces apoptosis in different cancers *via* multiple pathways, including P13-Akt-mTOR, VEGFR2, c-Met, PLCγ1, MAPT, JNK/p38, and ERK1/2, along with a combination of regulatory targets. For instance, LA induces apoptosis in HepG2 cells (hepatocellular carcinoma cell line) *via* mitochondrial dysfunction. This activity relies on the upregulation of mRNA expression of targets, such as DR3, DR5, caspase-3, caspase-8, caspase-10, Fas, Bad, Bax, Bcl-2, Bak, and PUMA. Downregulation of PKCε, p70S6K, and Akt is also described (Wang J. et al., 2018). Additionally, LA induced ER stress in HepG2 cells to induce apoptosis *via* activation of receptors of VEGFR2 and c-Met, phosphorylation of phospholipase Cγ1 (PLCγ1), and enhancing cytosolic Ca^2+^ release from the ER (Choi A. Y. et al., 2014). LA induced MCF-7 cell (breast cancer cell line) apoptosis at different autophagy and intrinsic pathways doses. Doses of 5–50 μM induced autophagy and apoptosis of MCF-7 by inhibiting PI3K-Akt-mTOR signaling, thereby increasing caspase-3 activity, decreasing expression of B-cell lymphoma-2, and triggering the release of cytochrome from mitochondria into the cytoplasm (Xue et al., 2018; Huang et al., 2019b). Lower doses (5–15 μM) activated intrinsic pathway-mediated apoptosis involving upregulation of Bax expression and PARP cleavage, downregulation of Bcl-2 and Cyclin D1, and accumulation of reactive oxygen species (ROS) (Buso Bortolotto et al., 2017).

As for lung cancer, LA induced apoptosis in A549 lung cancer cells by increasing the expression of apoptotic proteins and decreasing the expression of anti-apoptotic proteins (Lin Y. J. et al., 2019). Furthermore, relatively low doses (10–15 μM) induced apoptosis in non-small cell lung cancer (NSCLC) cells, such as A549 and H460 cells, by inducing ER stress. LA upregulated caspase-3 expression and increased PARP cleavage. CHOP expression was elevated in parallel (Tang et al., 2016; Qiu et al., 2017). Blocking the CHOP-mediated pathway inhibited the expression of downstream apoptotic proteins at a higher dose (40 μM) (Chen et al., 2018). LA significantly activated ERK and p38 in A549 and H460 cells in a time-dependent manner. LA also inhibited the activity of JNK, suppressed the expression of c-IAP1, c-IAP2, XIAP, Survivin, c-FLIPL, and RIP1, and attenuated LA-induced induction of autophagy (Luo et al., 2021). LA also promoted the degradation of EGFR, Met, Her2, and Akt through inactivating EGFR/Met-Akt signaling, resulting in apoptosis of gefitinib-resistant NSCLC cells (H1975) (Seo, 2013). Strikingly, LA was a protein synthesis inhibitor, which reduced both cap-dependent and independent translation. LA may inhibit the phosphorylation of 4EBP1 (Ser 65) and activate the PERK-eIF2α pathway to inhibit PD-L1 translation. Moreover, LA-induced ROS production in lung cancer cells is time-dependent (Yuan et al., 2021). LA thus displays a potential for use in cancer immunotherapy.

LA primarily induced apoptosis in bladder cancer cells through mitochondrial stress combined with ER stress. The former, observed at LA doses of 10–80 μM, induced T24 cells (bladder cancer cell line) apoptosis in a complex fashion. Mitochondrial membrane potential was decreased, and mRNA expression of Apaf-1, caspase-9, and caspase-3 was upregulated. These actions increased PARP cleavage and Bax/Bcl-2 ratio and increased intracellular Ca^2+^ level and ROS production. Detailed mechanisms of this latter activity require more in-depth studies (Yuan et al., 2013; Yang et al., 2016; Hong et al., 2019). LA also induced apoptosis in five gastric cell lines –GES-1, MKN-28, SGC7901, AGS, and MKN-45 (Xiao et al., 2011; Lin et al., 2017). LA induced BGC-823 cell apoptosis through ROS-mediated activation of MAPKs and PI3/AKT signaling at a higher dose (Hao et al., 2015). Hexokinase 2Â (HK2) expression was downregulated at a lower dose, attenuating glycolysis elevation and inducing apoptosis in MKN-45 and SGC7901 cells (Wu J. et al., 2018).

Moreover, LA induced apoptosis in oral squamous cell carcinoma with distinct mechanisms at different doses. In HN22 and HSC4 cells, LA induced apoptosis *via* downregulating the expression of specificity protein 1 (Sp1), upregulating Bax, Bid, Bcl-xl, caspase-3, and PARP cleavage with doses of 10–40 μM (Cho et al., 2014). In addition, LA induced apoptosis in KB cells, relying on activation of caspase-dependent factor associated suicide ligand (FasL) mediated death receptor pathway. This action induced upregulation of FasL and activation of caspase-8 and -3 along with PARP cleavage (Kim et al., 2014). In SCC-25 cells (oral squamous cell carcinoma cell line), LA (15–296 μM) induced both death receptor and mitochondrial apoptotic pathways *via* activation of caspase-3 (Zeng et al., 2014). Remarkably, LA showed an excellent ability to induce apoptosis in glioma stem cells (GS-Y01, GS-Y03, U87GS, GS-NCC01, and A172GS) at an attractive dosage (2–12.5 μM) by inducing mitochondrial fragmentation *via* activating of caspases-3, -8, and -9, combined with reducing mitochondrial membrane potential and inhibiting ATP production *in vitro* (Kuramoto et al., 2017).

The literature also showed that LA induced apoptosis and autophagy in SiHa (Tsai et al., 2015) and HeLa cervical cancer cells (Szliszka et al., 2012), human nasopharyngeal carcinoma cells (HONE-1, NPC-39, and NPC-BM) (Chuang et al., 2019), human pharyngeal squamous carcinoma FaDu cells (Yang et al., 2014; Park et al., 2015), OVCAR-3 and SK-OV-3 ovarian cancer cells (Lee et al., 2012; Kim et al., 2013), B16 melanoma cells (Chen et al., 2019), human osteosarcoma 143B cells (Lin R. C. et al., 2019), malignant pleural mesothelioma (MSTO-211H and H28 cells) (Kim K. H. et al., 2015).

### Effects of LA on Inhibiting Proliferation of Cancer Cells

Unlimited proliferation is a crucial feature of cancer cells. The pharmacological ability to inhibit proliferation is a crucial aspect of the anticancer effects of LA. Generally, inhibition of cancer cell proliferation reflects blocking the cell cycle at different transition phases *via* regulating specific mRNAs and protein levels, such as Wee1, P21, Cyclins, MDM2, Survivin, and CDK1. Occasionally, LA acts directly as a selective agonist or inhibitor involving JNK1, Ras homolog, R132C-mutant IDH1, and Sp1.

In detail, numerous studies indicated that LA blocked the cell cycle at the G2/M transition at different doses. For instance, LA blocked the cell cycle in HepG2 cells by increasing the expression of Weel, P21, Cyclin D1, and JNK1 and decreasing the expression of Survivin, Cyclin B1, and CDK1 using doses of 30–70 μM (Wang J. et al., 2018). Similarly, blocking at G2/M in NSCLC cells reflected downregulation of expression of MDM2, Cyclin B1, Cdc2, and Cdc25C at doses of 2.5–80 μM (Qiu et al., 2017). Also, LA stopped the cell cycle in T24 at the G2/M transition by downregulating the expression of Cyclin A, Cyclin B1, Wee1 and upregulating cyclin-dependent kinase (Cdk) inhibitor p21WAF1/CIP1 at a dose of 10–60 μM (Jiang et al., 2014; Hong et al., 2019). Cell cycle progression in MKN-28, MKN-45, and AGS cells (gastric cancer and colon cancer cell lines) was similarly blocked *via* upregulation of expression of retinoblastoma and downregulation of Cyclin A, Cyclin B, and MDM2 at doses of 5–50 μM (Xiao et al., 2011; Lin et al., 2017). Doses of approximately 10–40 μM blocked the U87 glioma cell cycle at G0/G1 and G2/M phases (Lu et al., 2018) and osteosarcoma HOS cells at G2/M transition (Shen et al., 2019). Additionally, LA significantly inhibited the expression of PD-L1 by blocking the interaction between p65 and Ras, thus inhibiting cell proliferation and promoting apoptosis (Liu et al., 2021).

LA also inhibits the proliferation of cancer cells directly by interaction with proteins and signaling. LA directly inhibited p38/JNK/ERK signaling in HepG2 cells and inhibited proliferation, and induced apoptosis in parallel (Chen X. et al., 2017). Furthermore, LA activated miR-142-3p to enhance the expression of Ras homolog enriched in the brain (Rheb) and subsequently activated mTOR signaling to inhibit proliferation, attenuate melanin production, and induce apoptosis in A375 and B16 melanoma cells (Barry and Parsons, 2016; Zhang et al., 2020). Another document indicated that LA inhibited Cyclin D1 expression resulting in cell cycle arrest at G1 in MCF-7 cells (Buso Bortolotto et al., 2017). In addition, LA (10–30 μM) also suppressed Sp1 to inhibit proliferation in MCF-7 and MDA-MB-231 cells (breast cancer cell lines) (Kang et al., 2017). Moreover, in different cell models, LA exhibited distinct oral squamous cell carcinoma mechanisms. In SCC-25 cells, LA inhibited proliferation by blocking the cell cycle at S and G2/M phases (Zeng et al., 2014), while in HN22 and HSC4 cells, proliferation was blocked by inhibiting Sp1 expression and downstream related proteins, including p27, p21, Cyclin D1, Mcl-1 and Survivin (Cho et al., 2014). As a selective inhibitor of R132C-mutant IDH1, LA inhibited the proliferation of sarcoma HT-1080 cells with an acceptable IC_50_ = 5.176 μM (Hu et al., 2020). Notably, 30 μmol/L LA treatment for 24 h significantly reduced cell viability of Arkansas P1 cells (ARP1) and Ontario Cancer Institute myeloma 5 cells (OCI-MY5) (Bai et al., 2021).

### Effects of LA on Inhibiting Migration and Invasion of Cancer Cells

Carcinoma cells own the ability to invade and migrate from their usual location to adjoining tissues and spread to other organs. Thus, inhibition of migration and invasion are essential aspects of anticancer activity. LA downregulated uPA expression in hepatocellular carcinoma by suppressing MKK4/JNK and NF-κB signaling and inhibited invasion and migration of HA22T/VGH and SK-Hep-1 cells (hepatocellular carcinoma cell models) at 5–20 μM (Tsai et al., 2014; Wu M. H. et al., 2018). Furthermore, 5–40 μΜ LA blocked the expression of E-cadherin and vimentin by suppressing MAPK and AKT signaling. This action inhibited invasion and migration in MDA-MB-231 cells (Huang et al., 2019b). Together with the AKT signaling pathway and downstream transcription factors Sp1 expression interruption, LA exhibited the ability to inhibit migration and invasion of A549 and H460 cells at relatively lower doses (2–20 μM) (Huang et al., 2014). LA inhibited oral squamous cell carcinoma proliferation, invasion, and migration *via* PI3K/AKT signaling (Hao et al., 2019).

### Other Aspects of Anticancer Effects

Apart from the therapeutic effects during carcinoma progression, LA also displays other anticancer activities for protecting cellular DNA, impairing pathogenicity, modulating the immune response, suppressing drug resistance, and attenuating vomiting.

As mentioned under *Effects of LA on Inducing Apoptosis of Cancer Cells*, LA enhanced cytosolic Ca^2+^ release from ER in HepG2 cells. This action further induced ROS accumulation and increased effective doses of cyclophosphamide, doxorubicin, vincristine, and prednisone (CHOP) (Choi A. Y. et al., 2014), thus improving the efficacy of these anticarcinogens. LA also reduced the efflux of doxorubicin and temozolomide from BCRP-mediated BCRP-MDCKII cells by downregulating the expression of breast cancer resistance protein (BCRP) (Fan et al., 2019). Based on DEGs and GO and KEGG enrichment, LA showed antitumor activity against HepG2 cells, in which MAPK and FoxO signaling played essential roles (Wang et al., 2021). In gefitinib-resistant non-small cell lung cancer cells (H1975), LA also reduced Hsp90 activity in gefitinib-resistant NSCLC cells (H1975) *via* binding to the *N*-terminal ATP binding site of Hsp90 to reduce drug resistance (Seo, 2013).

In addition to reducing drug resistance, low doses of LA (2–10 μM) enhanced the positive correlation between the activation of quinone oxidoreductase 1 (NQO1) and Keap1-Nrf2 signaling HepG2-ARE-C8 cells. LA protected cellular DNA from oxidative stress-induced damage and assisted the development of a prevention strategy against cancer (Hajirahimkhan et al., 2015; Li et al., 2015; Wang S. et al., 2018). Also, chemoproteomic profiling analysis suggested that LA could attenuate the pathogenicity of triple-negative breast cancers by inhibiting the production of prostaglandin reductase (Roberts et al., 2017). For bladder cancer, LA inhibited arginine methyltransferase (PRMT) activity in bladder cancer by targeting CARM1, thereby downregulating the expression of an epigenetic modification enzyme (Cheng and Bedford, 2011).

Furthermore, LA exhibited various effects *via* different mechanisms *in vivo*. For instance, the administration of LA (40 mg/kg) to C3H/HeN mice bearing UM-UC-3 cells enhanced the activity of cytotoxic T lymphocytes and counts of CD4^+^ CD25^+^ Foxp3^+^ T regulatory T cells. Thus, LA might treat bladder cancer by modulating the tumor immune microenvironment (Zhang et al., 2016). Administration of 10 mg/kg of LA attenuated the development of AngII-induced abdominal aortic aneurysm in mice through inhibiting expression of miR-181b, upregulating SIRT1/HO-1 signaling, and suppressing elastin degradation, matrix metalloproteinase and pro-inflammatory cytokines (Hou et al., 2019). Finally, LA inhibited 5-HT3 receptors, which are crucial mediators of nausea. Licorice has been used to treat stomach ulcers, gastrointestinal inflammation, and respiratory diseases (Lee et al., 2008). LA may be involved in regulating peristalsis and digestion to improve symptoms of functional dyspepsia and irritable bowel syndrome. In one study, licorice extract exhibited the strongest 5-HT3 inhibition of the tested extracts, and LA was identified as a partial antagonist of 5-HT3 receptors (Herbrechter et al., 2015).

With the literature in hand, we found that LA exhibited significant therapeutic properties in various carcinoma cells *via* various mechanisms *in vivo* and *in vitro* throughout the cancer progression. These properties include direct anticancer effects, such as inducing apoptosis and inhibiting proliferation, invasion, and migration. LA even displays other positive influences while maintaining therapeutic functions for cancers, including reducing drug resistance, impairing pathogenicity, modulating immune responses, and attenuating emesis (Table 1). Above all, LA as an adjunct to existing anticancer drugs may have broad applications. Hence, clinical anticancer research and in-depth investigation of the mechanisms of LA might be an important research direction.

**TABLE 1:** The anticancer effects of licochalcone A.

Cancer classification	Activity/mechanism(s) of action	Cell lines	Administration dosage	Application	References
Hepatocellular carcinoma	Induced apoptosis and inhibited proliferation	HepG2 cells	30–70 μM	*In vitro*	Wang et al. (2018a)
	Inhibited the PI3K-Akt-mTOR signaling pathway	HepG2 cells	5–20 μM	*In vitro*	Wu et al. (2019)
	Caused endoplasmic reticulum (ER) stress	HepG2 cells	1–50 μM	*In vitro*	Choi et al. (2014a)
	Activated the ULK1/Atg13 pathway	HepG2 cells	1–100 μM	*In vitro*	Niu et al. (2018)
	Inhibited the p38/JNK/ERK signaling pathway	HepG2 cells	24–95 μM (8–32 μg/ml)	*In vitro*	Chen et al. (2017b)
	Inhibited the migration and invasion	HA22T/VGH and SK-Hep-1 cells	5–20 μM	*In vitro*	Tsai et al. (2014); Wu et al. (2018b)
Breast cancer	Inhibited PI3K/Akt/mTOR and MAPK signaling pathways	MCF-7 and MDA-MB-231 cells	5–50 μM	*In vitro*	Xue et al. (2018); Huang et al. (2019b)
	Induced apoptosis	MCF-7 cells	15–44 μM (5–15 μg/ml)	*In vitro*	Buso Bortolotto et al. (2017)
	Inhibited the expression of specificity protein 1 (Sp1)	MCF-7 and MDA-MB-231 cells	10–30 μM	*In vitro*	Kang et al. (2017)
	Impaired the pathogenicity	Triple-negative breast cancers	1–100 μM	*In vitro*	Roberts et al. (2017)
	Reduced the efflux of doxorubicin and temozolomide	BCRP-MDCKII cells	IC_50_ ≈ 50 μM	*In vitro*	Fan et al. (2019)
	Induced the ER stress and block the cell cycle at the G2/M transition	NSCLC, A549 cells, and H460 cells	10–15 μM	*In vitro*	Tang et al. (2016); Qiu et al. (2017)
	Induced the ER stress	H292 cells	10 μM	*In vitro*	Chen et al. (2018)
	Suppressed the migration and invasion	A549 and H460 cells	2–20 μM	*In vitro*	Huang et al. (2014)
	Reduced drug resistance and induced apoptosis	Gefitinib-resistant non-small cell lung cancer cells	10–100 μM	*In vitro*	Seo, (2013)
Bladder cancer	Triggered mitochondrial dysfunction	T24 cells	10–80 μM	*In vitro*	Yang et al. (2016); Hong et al. (2019)
	Blocked cell cycle at G2/M transition	T24 cells	10–60 μM	*In vitro*	Jiang et al. (2014); Hong et al. 2019)
	Modulated the immune response	In C3H/HeN mice carrying UM-UC-3 cells	40 mg/kg	*In vivo*	Zhang et al. (2016)
Oral squamous cell carcinoma	Activated the PI3K/AKT signaling pathway	SCC4 and CAL-27 cells	25–100 μM	*In vitro*	Hao et al. (2019)
	Downregulated the expression of Sp1 and inhibited proliferation	HN22 and HSC4 cells	10–40 μM	*In vitro*	Cho et al. (2014)
	Induced apoptosis and inhibited the proliferation	SCC-25 cells	15–296 μM (5–100 μg/ml)	*In vitro*	Zeng et al. (2014)
	Inhibited the migration and invasion	SCC-25	74–296 μM (25–100 μg/ml)	*In vitro*	Shen et al. (2014)
Glioma	Induced mitochondrial fragmentation	GS-Y01, GS-Y03, U87GS, GS-NCC01, A172GS cells	2–12.5 μM	*In vitro*	Kuramoto et al. (2017)
	Inhibited proliferation and attenuated tumor growth in mice	U87 glioma cell and xenograft tumor mice	5–40 μM and 10 mg/kg, respectively	*In vitro* and *in vivo*	Lu et al. (2018)
	Inhibited the migration and invasion	M059K, U-251 MG, and GBM8901 cells	10–50 μM	*In vitro*	Huang et al. (2018)
Gastric cancer and colon cancer	Induced the apoptosis	GES-1, MKN-28, SGC7901, AGS and MKN-45 cells	IC_50_ = 92.7, 42.0, 40.8, 41.1 and 40.7 μM, respectively	*In vitro*	Xiao et al. (2011); Lin et al. (2017)
	Activated MAPKs and PI3K/AKT signaling pathways	BGC-823 cells	200–400 μM	*In vitro*	Hao et al. (2015)
	Downregulated the expression of HK2	MKN-45 and SGC7901 cells	15–60 μM	*In vitro*	Wu et al. (2018a)
	Blocked the cell cycle at the G2/M transition	MKN-28, AGS and MKN-45 cells	5–50 μM	*In vitro*	Xiao et al. (2011); Lin et al. (2017)
	Inhibited the proliferation and induced apoptosis	HCT116 cells and xenograft tumor mice	5–25 μM and 10–20 mg/kg	*In vitro*	Yao et al. (2014)
Cervical cancer	Inhibited the PI3K/Akt/mTOR signaling pathway	SiHa cells	10–50 μM	*In vitro*	Tsai et al. (2015)
	Increased the expression of TRAIL-R2	HeLa cells	25–50 μM	*In vitro*	Szliszka et al. (2012)
Ovarian cancer	Induced the apoptosis	OVCAR-3 and SK-OV-3 cells	5–25 μM	*In vitro*	Lee et al. (2012); Kim et al. (2013)
Oral squamous cell carcinoma	Activated the caspase-dependent factor-associated suicide ligand (FasL)-mediated death receptor pathway	KB cells	IC_50_ = 50 μM	*In vitro*	Kim et al. (2014)
Nasopharyngeal carcinoma	Activated the JNK/p38 signaling pathway	HONE-1, NPC-39, and NPC-BM cells	10–80 μΜ	*In vitro*	Chuang et al. (2019)
Pharyngeal squamous carcinoma	Activated the ERK1/2 and p38 MAPK signaling pathways	FaDu cells	IC_50_ = 100 µM	*In vitro*	Yang et al. (2014); Park et al. (2015)
Melanoma	Activated the expression of miR-142-3p	A375 and B16 cells	10–40 μM	*In vitro*	Barry and Parsons (2016); Zhang et al. (2020)
	Activated the mTOR signaling pathway	B16 cells	10–50 μM	*In vitro*	Chen et al. (2019)
Osteosarcoma	Activated the ATM-Chk2 checkpoint pathway and autophagy	HOS cells	10–40 μM	*In vitro*	Shen et al. (2019)
	Activated the p38MAPK pathway	143B cells and xenograft tumor mice	20–100 μM and 10 mg/kg	*In vitro* and *in vivo*	Lin et al. (2019a)
Abdominal aortic aneurysm	Inhibited the expression of miR-181b	AngII-induced abdominal aortic aneurysm in mice	5 and 10 mg/kg	*In vivo*	Hou et al. (2019)
Malignant pleural mesothelioma	Activated the mitochondrial apoptotic pathway	MSTO-211H and H28 cells	IC_50_ = 26 and 30 μM, respectively	*In vitro*	Kim et al. (2015a)
Sarcoma	Inhibited the R132C-mutant IDH1	HT-1080 cells	IC_50_ = 10.75 μM	*In vitro*	Hu et al. (2020)

## Anti-Inflammation Activities of LA

**FIGURE 4:**
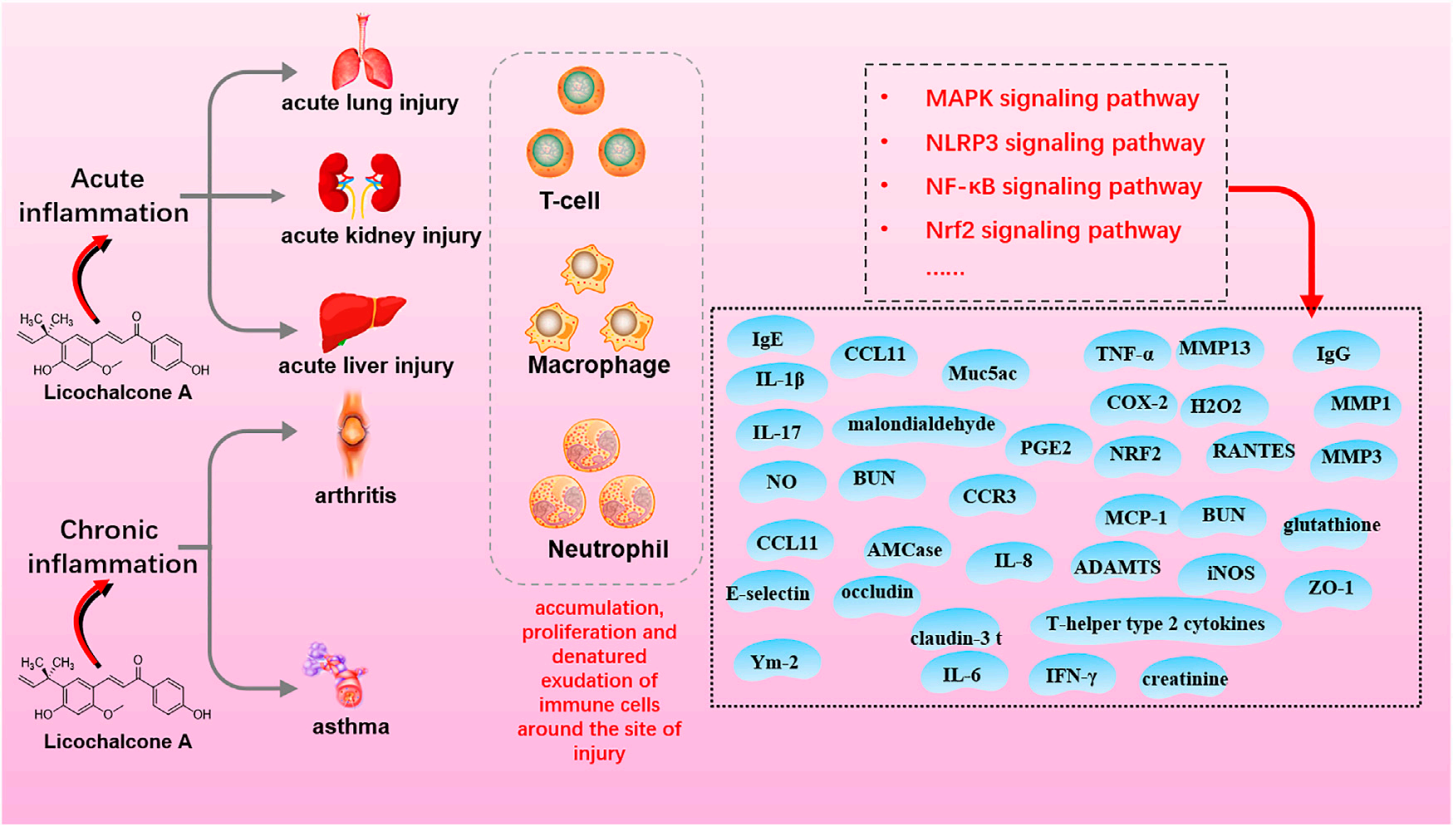
The mechanisms of licochalcone A with anti-inflammation activity.

Inflammation is an immune response to biological, physical, or chemical injury and foreign bodies or necrotic tissue. Inflammation is classified as acute or chronic based on the duration and intensity of inflammatory stimuli and mitigation *in situ*. LA demonstrates anti-inflammatory activity *via* interaction with MAPK, NF-κB, NLRP3, and Nrf2 signaling in the acute lung, kidney, and liver injury (acute inflammation) and arthritis and asthma (chronic inflammation) (Figure 4; Table 2).

**TABLE 2:** Anti-inflammation activities of LA.

Pharmacological effects	Activity/mechanism(s)	Cell lines/models	Administration dosage	Application	References
Anti-inflammatory	Inhibited the production of NO, TNF-α, IL-1β, IL-6, and PGE2	RAW 264.7 cells	5–20 μM	*In vitro*	Chu et al. (2012); Fu et al. (2013)
	Inhibited the NF-κB signaling pathway	A549 cells	1.5–30 μM	*In vitro*	Sulzberger et al. (2016)
	Inhibited MAPK and AKT/NF-κB signaling pathways	Mouse mammary epithelial cells	4–9 μM (1.2–3 μg/ml)	*In vitro*	Guo et al. (2019)
	Inhibited NF-κB and p38/ERK MAPK signaling pathways	Acute lung injury mice	20–80 mg/kg	*In vivo*	Chu et al. (2012); Liu et al. (2019)
	Inhibited the NF-κB signaling pathway	Acute kidney injury mice	20–80 mg/kg	*In vivo*	Hu and Liu, (2016)
	Inhibited TLR4-MAPK and -NF-κB and Txnip-NLRP3 signaling pathways	Acute lung injury mice	100 mg/kg	*In vivo*	Lv et al. (2019)
	Inhibited NF-κB and activated the Nrf2 signaling pathways	Colitis mice	20–80 mg/kg	*In vivo*	Liu et al. (2018)
	Inhibited pro-inflammatory factor production and regulated the immune response	Encephalomyelitis mice	30 mg/kg	*In vivo*	Almeida Fontes et al. (2014a); Almeida Fontes et al. (2014b)
	Inhibited NF-κB and wnt/β-catenin signaling pathways	Mice chondrocytes	5–20 μM	*In vitro*	Sulzberger et al. (2016); Chen et al. (2017a); Jia et al. (2017)
	Activated the Keap1-Nrf2 signaling pathway	Arthritis mice	25–50 mg/kg	*In vivo*	Su et al. (2018)
	Inhibited the NF-κB signaling pathway	Arthritic mice	25–50 mg/kg	*In vivo*	Yang et al. (2018)
	Inhibited the IKK/NF-κB/TSLP signaling pathway	BEAS-2B cells	10–30 μM	*In vitro*	Kim et al. (2015b)
	Inhibited VEGFR2 and ERK1/2 signaling pathways	Mouse airway smooth muscle cells	10–30 μM	*In vitro*	Kim et al. (2017)
	Inhibited pro-inflammatory and allergenic factor expression	Allergic asthma mice	5–50 mg/kg	*In vivo*	Chu et al. (2013); Huang et al. (2019)
	Inhibited CD3 and CD28 antibody-induced T-cell IL-2 secretion	HEK293T, RBL-2H3, Jurkat T-cells	2.97 ± 1.217 μM, 0.83 ± 1.222 μM, 11.21 ± 1.07 μM, respectively	*In vivo*	Phan et al. (2021)

### Effects of LA on Acute Inflammation

Acute inflammation is a protective response characterized by the rapid accumulation of immune cells around an injury site. The response features short duration, local edema, and leukocyte emigration. LA exerts anti-inflammatory effects in a dose-dependent manner. LA, 20–80 mg/kg, inhibited NF-κB signaling during treatment of acute inflammation in various mouse models, such as LPS-induced acute lung injury (Chu et al., 2012; Liu et al., 2019), LPS-induced acute kidney injury (Hu and Liu, 2016), and DSS-induced colitis (Liu et al., 2018). In detail, NF-κB and p38/ERK MAPK signaling pathways were inhibited in LPS-induced acute lung injury, and LA decreased the number of inflammatory cells, lung wet-to-dry weight ratio, protein leakage, and myeloperoxidase activity (Chu et al., 2012; Liu et al., 2019). LA also reduced the production of TNF-α, IL-6, and IL-1β, and serum BUN and creatinine *levels via* inhibition of LPS-induced NF-κB activation in LPS-induced acute kidney injury (Hu and Liu, 2016). In addition, LA effectively alleviated DSS-induced colitis by downregulating the expression of pro-inflammatory factors and oxidative stress *via* inhibiting NF-κB and activating Nrf2 signaling (Liu et al., 2018). Additionally, LA (30 mg/kg) improved clinical symptoms of myelin oligodendrocyte glycoprotein peptide-induced mouse encephalomyelitis by inhibiting H_2_O_2_, NO, IFN-γ, TNF-α, and IL-17 production and modulating the immune response of Th1 and Th17 cells (Almeida Fontes L. B. et al., 2014; Almeida Fontes LB. et al., 2014). At a higher dose (100 mg/kg), LA attenuated LPS-induced mouse acute liver injury *via* reducing pro-inflammatory cytokines, TNF-α, IL-6, and IL-1β, by inhibiting TLR4-MAPK-NF-κB and Txnip-NLRP3 signaling (Lv et al., 2019).

Furthermore, *in vitro* studies suggested mechanisms of LA in acute inflammation treatment consistent with *in vivo* data. LA inhibited the production of NO, TNF-α, IL-1β, IL-6, and PGE2 in LPS-induced RAW 264.7 macrophages at doses of 5–20 μM (Chu et al., 2012; Fu et al., 2013). In addition, LA blocked MAPK, and AKT/NF-κB signaling increased protein levels of ZO-1, occludin, and claudin-3, and exerted an anti-inflammatory effect on LPS-induced mouse mammary epithelial cells at doses of 4–9 μM (Guo et al., 2019). As for TNF-α-induced A549 cells (acute lung injury model), LA suppressed the inflammatory response in TNF-α-induced A549 cells (acute lung injury model) by inhibiting NF-κB signaling and PGE2 secretion at doses of 1.5–30 μM (Sulzberger et al., 2016).

### Effects of LA on Chronic Inflammation

Chronic inflammation is a slow, long-term process that varies by etiology and repair capacity. Acute inflammation tends to develop into chronic inflammation due to the persistence of inflammation or poor repair of damage. The ubiquity of chronic inflammation-mediated diseases has attracted many researchers. Investigation of anti-inflammatory properties of LA on chronic inflammation has used arthritis and asthma models that represent typical chronic inflammation conditions. LA showed anti-inflammation activity on IL-1β-stimulated mouse chondrocytes in arthritis models by a complex mechanism involving inhibition of phosphorylation of NF-κBp65 and IκBαn, decreasing the production of PGE2 and NO, and downregulating the expression of iNOS, COX-2, ADAMTS, MMP1, MMP3, and MMP13 by blocking NF-κB and wnt/β-catenin signaling (Chen WP. et al., 2017).

Conversely, LA (25–50 mg/kg) inhibited secretion of pro-inflammatory cytokines and upregulation of antioxidant enzyme expression *via* the Keap1-Nrf2 signaling pathway to reduce inflammation in collagen antibody-induced arthritic mice *in vivo* (Su et al., 2018). What is more, LA altered the morphology, ultrastructure, and stiffness of synovial fibroblast membranes in mouse rheumatoid arthritis. The agent also decreased paw swelling in antigen-induced mice by inhibiting IκBα phosphorylation and degradation and p65 nuclear translocation and phosphorylation (Yang et al., 2018).

As for asthma models, LA improved symptoms in animal asthma models and slowed the progression of airway inflammation. The study evidenced that LA suppressed the expression of thymic stromal lymphopoietin and other pro-inflammatory mediators, such as MCP-1, RANTES, and IL-8, in polyinosinic-polycytidylic acid-induced BEAS-2B cells and primary bronchial epithelial cells with doses of 10–30 μM (Kim SH. et al., 2015) through inhibition of KK/NF-κB/TSLP signaling. LA also inhibited VEGF-induced mouse airway smooth muscle cell proliferation at the above doses *via* blocking VEGFR2 and ERK1/2 signaling and downregulating the expression of caveolin-1 (Kim et al., 2017). Additionally, LA (5–50 mg/kg) reversed the progression of airway inflammation in OVA-induced allergic asthmatic mice by inhibiting the expression of Ym-2, AMCase, Muc5ac, E-selectin, CCL11, CCR3, and T-helper type 2 cytokines, simultaneously with dynamic regulation of levels of malondialdehyde, IgE, IgG, and glutathione (Chu et al., 2013; Huang et al., 2019a). LA inhibited ORAI1, Kv1.3, and KCa3.1 channels and significantly inhibited the production and secretion of IL-2 in T cells induced by CD3 and CD28 antibodies *in vivo*. Overall, LA may offer an effective therapeutic strategy for inflammation-related immune diseases (Phan et al., 2021).

## Other Pharmacological Effects and Pharmacokinetic Details

LA also exhibits additional pharmacological properties. Activities include antibacterial (Hao et al., 2013; Shen et al., 2015; Michelotto Cantelli et al., 2017), antioxidant (Su et al., 2018), bone protection (Kim et al., 2012; Ming et al., 2014; Shang et al., 2014), anti-parasitic (Yadav et al., 2012; Tadigoppula et al., 2013), neuroprotection (Lee SY. et al., 2018), skin protection (Angelova-Fischer et al., 2014; Angelova-Fischer et al., 2018), regulation of blood glucose and blood lipids (Quan et al., 2012; Quan et al., 2013; Lee HE. et al., 2018). Cellular processes are primarily associated with inflammatory responses, lipid metabolism, and oxidative stress. Signaling pathways involved include NF-κB and sirt-1/AMPK, and Nrf2 oxidative stress (Sulzberger et al., 2016; Liu et al., 2018; Lv et al., 2019). In an *in vivo* pharmacokinetic study after oral administration of LA to SD rats (200 mg/kg), Cmax was 3.31 ± 0.30 μg/ml, AUC0-24 h was 21.83 ± 1.44 μg/ml, T1/2 was 7.21 ± 0.37 h, MRT was 10.26 ± 1.01 h (Zhu et al., 2021). Furthermore, Liu and others found a Cmax of 3.39 ± 0.22 μg/ml, AUC0-24 h of 19.37 ± 0.56 μg/ml, T1/2 value of 7.43 ± 0.51 h, MRT of 11.84 ± 0.67 h, and Tmax of 2 ± 0 h. Meanwhile, LA is rapidly distributed to all tissues, particularly in the liver, kidney, and spleen, within a short time (Choi JS. et al., 2014).

## Summary and Perspectives

We found that LA exhibits various beneficial pharmacological properties. First, LA demonstrates promising anticancer activity on epithelial carcinoma and mesenchymal sarcoma cells through apoptosis and ER stress processes. These biological processes involve several signaling pathways, such as PI3K/Akt/mTOR, P53, NF-κB, P38, and caspase-3 apoptosis that in turn involve multiple targets, such as TNF-α, VEGF, Fas, PI3K, AKT, caspase-3, caspase-8, caspase-4, caspase-9, and caspase-10. Moreover, LA displays a broad spectrum of pharmacological activities, such as anti-inflammation, antioxidant, antibacterial, anti-plasmodial, and positive blood glucose and lipids regulation. These activities are associated with cellular inflammatory responses, lipid metabolism, and oxidative stress. These processes are regulated by signaling pathways, such as NF-κB, sirt-1/AMPK, and Nrf2 oxidative stress.

Significant progress has been made over the last decade, but several challenges still hamper in-depth mechanism research and clinical trials. First, current studies of LA focus mainly on signaling pathways and proteins or biomolecules at the molecular level. DNA and RNA molecules also play essential roles in developing complex diseases (Michalak et al., 2019; Varshney et al., 2020). Hence, exploration of LA targeting disease-related DNA and RNA fragments could be a promising direction for research in the future. Second, during data collection, we found that LA could counter the intestinal toxicity caused by irinotecan (Song et al., 2021), and LA derivatives and artemisinin demonstrated a synergism that enhanced antimalarial effects (Sharma et al., 2014). Clinical research with drug or other bioactive compound combinations with LA could prove rewarding; this approach is consistent with LA’s role as a guiding medicinal herb in traditional Chinese formulas. Third, the development of computer science and AI technology accelerates the speed of drug discovery (Chan et al., 2019; Yang et al., 2019), and well-established organic synthetic technology enables us to rapidly and precisely synthesize desirable molecules derived from bioactive natural compounds (Zhuang et al., 2017; Afanasyev et al., 2019). Suitable modification of LA structure for production of novel derivatives (Boström et al., 2018), or precise connection of LA with a linker and ligand of E3 (ubiquitin ligase) for assembly of proteolysis targeting chimeras (Chamberlain and Hamann, 2019; Burslem and Crews, 2020), could lead to the discovery of LA derivatives with excellent therapeutical properties (Thomford et al., 2018). We hope this review provides an updated and reliable insight into scientific research related to LA.
